# The involvement of interleukin-22 in the expression of pancreatic beta cell regenerative *Reg* genes

**DOI:** 10.1186/2045-9769-2-2

**Published:** 2013-04-04

**Authors:** Thomas Hill, Olga Krougly, Enayat Nikoopour, Stacey Bellemore, Edwin Lee-Chan, Lynette A Fouser, David J Hill, Bhagirath Singh

**Affiliations:** 1_11 Department of Microbiology and Immunology, University of Western Ontario, London, ON Canada; 2_11 Inflammation and Immunology, Biotherapeutics Research and Development, Pfizer, Cambridge, MA 02140, USA; 3_11 Department of Physiology and Pharmacology, University of Western Ontario, London, ON Canada; 4_11 Lawson Health Research Institute, London, ON Canada; 5_11 Centre for Human Immunology, University of Western Ontario, London, ON Canada; 6_11 Robarts Research Institute, University of Western Ontario, London, ON Canada

**Keywords:** Adjuvant immunotherapy, Interleukin-22, Regenerating (Reg) genes, Beta-cell regeneration, Type 1 diabetes

## Abstract

**Background:**

In Type 1 diabetes, the insulin-producing β-cells within the pancreatic islets of Langerhans are destroyed. We showed previously that immunotherapy with Bacillus Calmette-Guerin (BCG) or complete Freund’s adjuvant (CFA) of non-obese diabetic (NOD) mice can prevent disease process and pancreatic β-cell loss. This was associated with increased islet Regenerating (*Reg*) genes expression, and elevated IL-22-producing Th17 T-cells in the pancreas.

**Results:**

We hypothesized that IL-22 was responsible for the increased *Reg* gene expression in the pancreas. We therefore quantified the *Reg1, Reg2,* and *Reg*3δ (INGAP) mRNA expression in isolated pre-diabetic NOD islets treated with IL-22. We measured *IL-22,* and *IL-22 receptor(R)-*α mRNA expression in the pancreas and spleen of pre-diabetic and diabetic NOD mice. Our results showed: 1) *Reg1* and *Reg2* mRNA abundance to be significantly increased in IL-22-treated islets *in vitro*; 2) IL-22 mRNA expression in the pre-diabetic mouse pancreas increased with time following CFA treatment; 3) a reduced expression of *IL-22R*α following CFA treatment; 4) a down-regulation in *Reg1 and Reg*2 mRNA expression in the pancreas of pre-diabetic mice injected with an IL-22 neutralizing antibody; and 5) an increased islet β-cell DNA synthesis *in vitro* in the presence of IL-22.

**Conclusions:**

We conclude that IL-22 may contribute to the regeneration of β-cells by up-regulating Regenerating *Reg1* and *Reg2* genes in the islets.

## Background

Type 1 diabetes (T1D), also known as juvenile diabetes, is an autoimmune disease characterized by the destruction of insulin-producing β-cells within the pancreatic islets of Langerhans. Type 1 diabetes is thought to be caused by a complex interaction between environmental and genetic factors that is still not fully understood [1]. This interaction causes initial damage to the pancreatic islets leading to a T-cell mediated autoimmune response that induces dysregulation and destruction of β-cells via the release of inflammatory cytokines [2]. Current T1D management includes insulin therapy and pancreas or islet cell transplantation; however, none of these procedures will ensure the complete removal of diabetic complications. Therefore, studying the endogenous regeneration of pancreatic β-cells may suggest strategies for alternative and long-lasting approaches for T1D management [3].

Endogenous β-cell regeneration is thought to occur either by whole islet neogenesis (WIN) via the differentiation of progenitor cells within the adult pancreas, or by β-cell replication (BCR) and the regeneration of new β-cells from pre-existing β-cells [3, 4]. Using non-obese diabetic (NOD) or streptozotocin-injected C57/BL6 mice as a model for T1D, several groups have shown a possible role for transcription factors, growth factors, and regeneration genes in stimulating β-cell regeneration. This could result in whole islet neogenesis (WIN) and subsequent insulin production and diabetes reversal. Some of these candidate factors include pancreatic and duodenal homeobox 1 (PDX-1), glucagon-like peptide-1 (GLP-1), islet neogenesis-associated protein (INGAP) and Regenerating protein 1 and 2 (Reg1 and Reg2) [3, 5–9]. Recently, platelet-derived growth factor (PDGF) has also been shown to stimulate β-cell regeneration via activating cyclin D1 and inducing a G1 to S transition of the β-cell cycle [10].

Previously, we have shown that a single injection of *Mycobacterium-*containing Complete Freund’s Adjuvant (CFA) into NOD mice has a protective effect against T1D by down- regulating autoimmunity and restoring normoglycemia via the induction of various regulatory T (Treg) cells [7, 11, 12]. The role of CFA on endogenous β-cell replenishment, identified by histological analysis, however, still remains controversial and the exact mechanism is presently unknown [5, 7].

Several groups have identified the Regeneration gene family (*Reg)* as being expressed during the process of WIN and β-cell regeneration in the pancreas [3, 5, 7, 9, 12]. There are seven types of *Reg* genes present in the mouse (located on chromosome six with the exception of *Reg*4) but only five have been shown to be present in the human (located on chromosome 2) [13]. The Reg proteins encoded by these genes are C-type lectins, and have been found to be also expressed in a variety of tissues such as the liver, kidney, brain, and gastrointestinal tissues [3, 13]. Once secreted, these soluble proteins act in an autocrine and/or paracrine manner to exert their effects on their cognate receptors, where they may stimulate an anti-microbial, anti-inflammatory, anti-apoptotic, or regenerative response depending on the tissue type [3, 7, 9, 13]. In the last decade, *Reg*1 and *Reg*3δ (INGAP), expressed by β-cells and acinar cells respectively, have been shown to be linked with pancreatic β-cell regeneration in the mouse by activating cyclin D1 and promoting β-cell cycle progression [9, 13, 14]. More recently, we have shown that *Reg2* is substantially up-regulated in the pancreatic islets, particularly in the β-cells, following adjuvant treatment in diabetic and non-diabetic NOD female mice, as well as in C57BL/6 mice treated with streptozotocin (STZ). This increased *Reg2* expression has been shown to be associated with an increase in insulin production, a partial reversal of insulitis, and an improved glucose tolerance test in STZ-treated diabetic C57BL/6 mice [5]. This led to the conclusion that β-cell regeneration via up-regulation of the *Reg2* gene may have the capacity to reverse T1D in diabetic mice immunized with CFA using a pathway that is similar to Reg1 and Reg3δ [5, 7].

CFA immunization has been shown to induce CD4+ Th17 T cells to produce interleukin IL-17, IL-22, IL-10 and IFN-γ in NOD mice [15]. This finding leads to the concept that adjuvant-induced cytokines may have the potential to activate transcription factors that stimulate Reg proteins such as Reg1, Reg2, and Reg3β (PAP1) [7]. Among these cytokines, IL-22 has been of specific interest because it is released by CD4+ Th17 T cells in diseases such as hepatitis and inflammatory bowel disease, where IL-22 expression levels have been shown to promote cell regeneration and survival in hepatocytes and colonal epithelial cells. Interestingly, IL-22 has also been found recently to induce *Reg* gene expression in the pancreatic acinar cells surrounding pancreatic islets [16]. IL-22 is a member of the IL-10 family of cytokines and the gene is located on human chromosome 12. Like IL-17A and IL-17F, IL-22 is a glycoprotein, which binds to its receptor complex (composed of IL-10Rβ and IL-22Rα) as a homodimer [17, 18]. The IL-22 receptor complex is highly expressed in the pancreatic α and β cells [19]. Upon receptor binding, the tyrosine kinases, JAK and Tyk1 are phosphorylated which leads to the activation of the STAT3 transcription factor and subsequent up-regulation of the *Reg* genes in the mouse and human [17, 18]. However, the possible induction of the *Reg* genes via IL-22 in the pancreatic islets or NOD mouse model has not yet been examined.

This study sought to test the effects of IL-22 on the regulation of the pancreatic *Reg* genes, with a focus on *Reg*2, in diabetic and pre-diabetic NOD female mice immunized with CFA. This was accomplished in a two-step process: Firstly, by studying the direct effect of IL-22 on *Reg* gene up-regulation in the NOD mouse pancreatic islets; and secondly by examining whether CFA immunization could up-regulate *IL-22* expression in the whole pancreas. It was hypothesized that CFA immunization in pre-diabetic mice would lead to the production of IL-22 via the induction of Th17 cells, and that the resulting IL-22 cytokine would activate a JAK-STAT3 signal transduction pathway following binding to its receptor complex on pancreatic β-cells. IL-22 signaling would then result in the up-regulation of *Reg* gene expression that may be linked to β-cell regeneration and the reversal of hyperglycemia in T1D. This, however, requires inhibition of autoimmunity to prevent the reversal of disease. Immunotherapies with mycobacterial adjuvants such as BCG vaccination have the potential to achieve both these objectives [20].

## Results

### Recombinant IL-22 up-regulates Reg2 and Reg1 mRNA expression in the pancreatic islets

**Figure 1:**
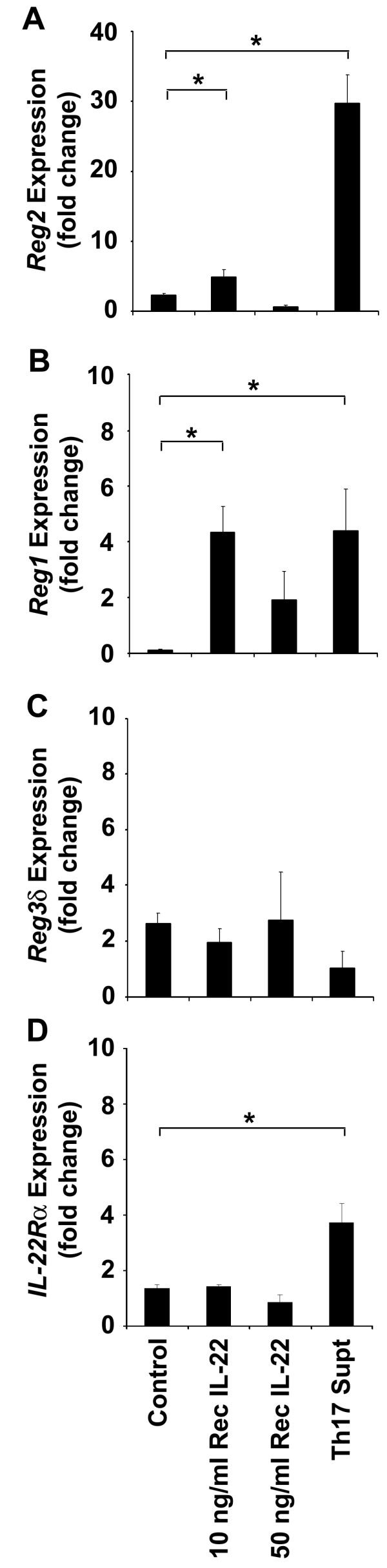
**Reg genes and IL-22 receptor** ***(IL-22*** **R** **α** **) expression in the pancreatic islet cells after IL-22 treatment.** Quantitative RT-PCR analysis was performed using gene-specific primers (Table 1) for *Reg*1, *Reg*2, *Reg*3δ *and IL-22*Rα on total RNA isolated from 6-week-old pancreatic islets (***A***-***D***). Islets were harvested 48 hrs after incubation with either DMEM medium (control); 10, or 50 ng/mL of recombinant IL-22; or a supernatant of Th17 cells polarized with IL-6 plus IL-23 (2 ml). Results shown represent the average fold-change in mRNA expression ± SEM when compared to control-treated islets. N = 3 mice (12 – 14 islets per mouse). Means indicated by the asterisk (*) are significantly different (P<0.05).

To establish whether IL-22 induces *Reg* gene expression, 6-week-old pre-diabetic NOD mouse islets were isolated and incubated with recombinant IL-22 at 10 and 50 ng/mL concentrations or supernatants from Th17 polarized splenocytes containing IL-17 and IL-22 [15] (Figure 1). Quantitative RT-PCR was performed on total extracted RNA from the treated pancreatic islets. *Reg*2 and *Reg*1 mRNA levels were found to be significantly higher, by approximately 3 and 4.2-fold respectively, in islets incubated with 10 ng/mL of IL-22 when compared to the media control (P<0.05) (Figure 1A and 1B). Interestingly, the higher 50 ng/mL dose of IL-22 significantly down-regulated *Reg*2 and *Reg1* expression (P<0.05). This was not due to the toxicity of the cytokine as there was no change in the viability of the treated islets cells as compared to media control. The highest fold increase in *Reg*2 gene expression was observed in islets treated with the supernatants of 4 days culture of BDC2.5 NOD splenocytes conditioned with IL-23 and IL-6 (Figure 1A). The supernatants also contained IL-22 and produced a significant 27-fold increase in *Reg2* mRNA abundance when compared to the media control (P<0.05). Islets treated with the Th17 T cell supernatant also showed a significant up-regulation of *Reg*1 gene expression by approximately 4.3-fold, when compared to the control islets (P<0.05).However, unlike Reg2, this Reg1 expression was not significantly different when compared to islets treated with 10 ng/mL of recombinant IL-22. No significant change in *Reg*3δ expression was observed in response to IL-22 or the Th17 T cell supernatant (Figure 1C).

The effect of recombinant IL-22 and the Th17 cell supernatant on IL-22 receptor expression was also tested on the same islet samples using gene-specific primers for the IL-22 receptor chain, IL-22Rα. As shown in Figure 1D the mean mRNA levels for *IL-22R*α did not increase in islets treated with 10 ng/ml of recombinant IL-22 and in fact was further reduced with 50 ng/ml of IL-22. Islets treated with the Th17 cell supernatant induced a significant 2.4-fold increase in IL-22Rα expression when compared to control-treated islets (control [1.36±0.21], Th17 T cells [3.72±0.98], P<0.05).

### CFA treatment increases the relative mRNA expression for IL-22 in pancreatic tissue

**Figure 2:**
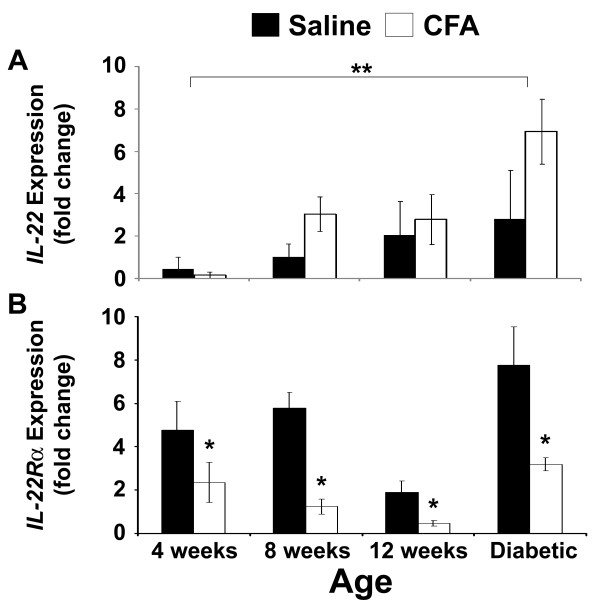
**Expression of** ***IL-22*** **and** ***IL-22*** **receptor** ***(IL-22*** **R** **α** **) in the pancreatic islet cells after CFA immunization.** The relative mRNA expression levels of *IL-22* and *IL-22*Rα (***A*** and ***B***) was carried out in the pancreas of non-diabetic (4-week old), pre-diabetic (8-week old and 12-week old), and older (>16 week-old) diabetic NOD mice following CFA immunization. Female NOD mice were injected i.p. with either 100 μl of CFA emulsified in saline or with saline alone and sacrificed 48 hrs following immunization. Whole pancreatic tissue was homogenized and total RNA extracted for reverse transcription quantitative real-time PCR analysis using gene-specific primers. The results shown for each treatment and age group have been compared to 4-wk-old saline-treated mice and represent the average fold change of mRNA expression ± SEM. The relative expression of mRNA was taken from three pooled samples of RNA per group (with three mice per pooled sample). Age groups indicated by a double asterisk (**) are significantly different (P<0.05); treatments indicated by the asterisk (*) are significantly different from the saline control (P<0.05).

We have previously shown that CFA injection in non-diabetic and diabetic NOD mice results in a substantial increase in *Reg* gene expression [5]. To determine whether the development of diabetes was accompanied by similar changes in *IL-22* gene expression in vivo, qRT-PCR analysis was performed using IL-22 gene-specific primers for cDNA using the various NOD mouse treatment/age groups. As shown in Figure 2A a significant effect of age on *IL-22* expression was detected (F= 6.374; *df*= 3; P=0.021). Post-hoc analysis revealed *IL-22* mRNA abundance in the pancreas to be significantly higher in diabetic mice when compared to non-diabetic (4-week-old) animals (P<0.05). The mean IL-22 expression tended to be higher in the pre-diabetic (8-week-old) mice and in diabetic mice following CFA treatment (by approximately 2 and 4-fold respectively).

### CFA treatment down-regulates the pancreatic expression of the IL-22 receptor sub-unit, IL-22Rα

It has previously been reported by Shioya *et al.*[19] that the IL-22Rα chain, a member of the IL-22 receptor complex, is specifically expressed in the α- and β-cells within the human pancreas. To determine whether CFA treatment affected the relative mRNA expression levels of *IL-22Rα* in the NOD mouse pancreas, qRT-PCR analysis was performed using total RNA extracted from non-diabetic (4-week-old), pre-diabetic (8-week-old and 12-week-old) and diabetic mice (Figure 2B). A significant interaction was identified between mouse age and treatment with CFA (F=6.146; *df*=3; P=0001). When analyzing the variables separately, CFA treatment was found to significantly down-regulate *IL-22R*α mRNA expression when compared to the saline-treated controls for all mouse ages (F=82.411; *df*=3; P<0.001) (Figure 2B). *IL-22Rα* mRNA expression was also found to significantly alter with age (F=24.212; *df*=3; P<0.001). Post-hoc analysis revealed *IL-22Rα* mRNA abundance to be significantly higher in diabetic mice (P<0.05), when compared to non-diabetic and pre-diabetic mice. Pre-diabetic mice at 12-weeks of age were found to have an *IL-22R*α mRNA abundance that was significantly lower (P<0.05), when compared to non-diabetic (4-week-old), pre-diabetic (8-week-old), and diabetic animals.

### Reg2 and Reg1 expression is down-regulated in CFA-treated mice following treatment with an IL-22 neutralizing antibody

**Figure 3:**
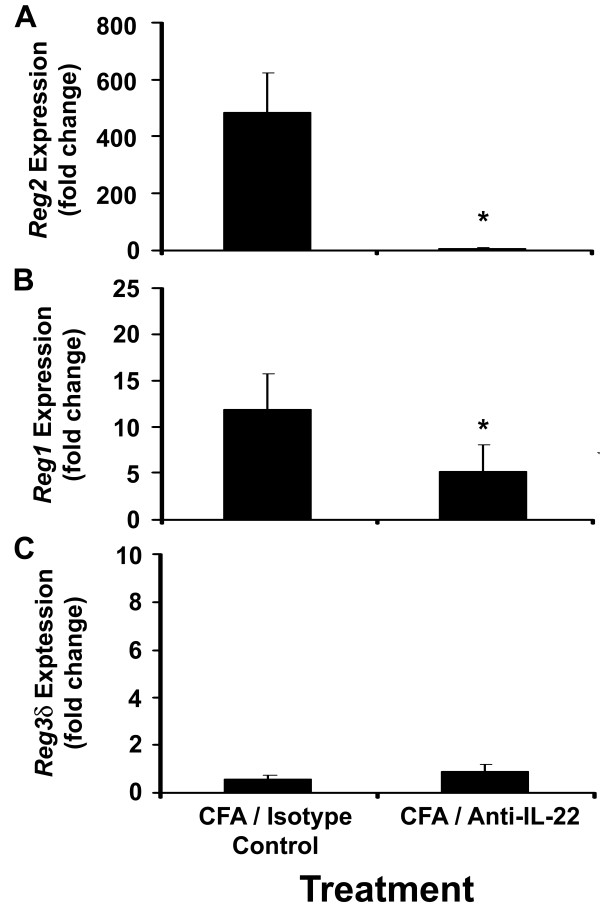
**Expression of Reg genes in the pancreatic islet cells after IL-22 neutralization.** *Reg1, Reg2*, and *Reg3*δ gene expression levels (***A***-***C***) was determined in the pancreas of 6-week-old NOD mice immunized with CFA and treated with an IL-22 neutralizing or a control antibody. Female NOD mice were injected i.p. with either 100 μl of CFA emulsified in saline followed by 100 μl of IL-22 neutralizing antibody (IL-22-01) 1 hr later, or 100 μl of CFA emulsified in saline followed by 100 μl of isotype control antibody. Mice were sacrificed 48 hrs following immunization. Whole pancreatic tissue was homogenized and total RNA extracted for reverse transcription quantitative real-time PCR analysis using gene-specific primers. The results shown for each treatment have been compared to mice immunized with CFA and the IgG isotype antibody, and represent the average fold change of mRNA expression ± SEM. The relative expression of mRNA was taken from three pooled samples per group (with three mice per pooled sample). Treatments indicated by the asterisk (*) are significantly different from the CFA control (P<0.001).

To determine whether IL-22 was directly involved in the up-regulation of *Reg* expression following CFA treatment, the abundance of *Reg2, Reg*1and *Reg*3δ mRNAs were measured in the pancreas of 6-week-old NOD mice immunized with CFA followed by i.p injection of an IL-22 neutralizing antibody 1 h later. Mice immunized with CFA and treated with the IL-22 neutralizing antibody [21] were found to have a significant reduction (approximately 480-fold) in *Reg*2 gene expression when compared to mice immunized with CFA and treated with an isotype control antibody (P<0.001) (Figure 3A). Likewise, *Reg*1 gene expression was significantly down-regulated in mice injected with the IL-22 neutralizing antibody (P<0.001) (Figure 3B). In contrast, however, no change in Reg3δ expression was observed for mice immunized with CFA in the presence of IL-22 neutralizing antibody when compared to mice injected with the isotype control antibody (Figure 3C).

### IL-22 and Reg mRNA expression in the spleen

**Figure 4:**
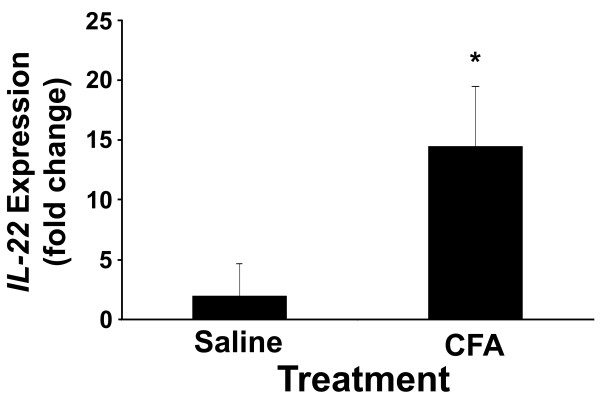
**Expression of** ***IL-22*** **in the spleen of 4-week-old non-diabetic NOD mice following CFA-treatment.** The relative mRNA expression of *IL-22* in the spleen of 4-week-old NOD mice was carried out following CFA-treatment. Quantitative RT-PCR analysis was performed using gene-specific primers (Table 1) on total RNA isolated from whole splenic tissue from female NOD mice injected i.p. with either 100 μl of CFA emulsified in saline or saline alone. Results shown represent the average fold-change in mRNA expression ± SEM compared with saline-treated mice. Results were taken from 3 mice per treatment. The asterisk (*) denotes a significant difference from the saline control (P<0.05).

Since the spleen is a major peripheral lymphoid tissue, we performed quantitative RT-PCR analysis on splenocytes derived from 4-week-old, pre-diabetic NOD mice, with or without CFA treatment, to investigate the presence of IL-22-producing Th17 cells during autoimmune insulitis (Figure 4). CFA treatment was found to significantly up-regulate *IL-22* expression in the non-diabetic, 4 week-old mice by approximately 12-fold (P<0.05). To determine whether CFA-mediated *Reg* gene up-regulation occurred in the spleen as well as in pancreatic tissue, *Reg1, Reg2* and *Reg3*δ mRNA expression was also analyzed in whole splenic tissue using non-diabetic mice with/without CFA injection. Similar to Huszarik *et al.*[5], no effect of treatment was detected on *Reg* mRNA expression (data not shown).

### CFA treatment does not change Reg or IL-22 mRNA abundance in the immune-deficient NOD.Scid mouse pancreas

To verify the finding by Huszarik *et al.*[5] that *Reg* up-regulation following CFA treatment is mediated by an infiltrating T cell response, qRT-PCR analysis was performed on the pancreas of CFA treated non-diabetic (4-week-old) NOD.Scid mice. NOD-severe combined immune-deficient (Scid) mice have a deleterious single nucleotide polymorphism (SNP) in the *Prkdc* gene that ultimately affects T- and B- lymphocyte development. As reported by Huszarik *et al.*[5], we found no increase in *Reg*1, *Reg2*, or *Reg3*δ mRNA abundance in the pancreas of NOD.Scid mice following CFA treatment (data not shown). This procedure was repeated using IL-22 gene-specific primers to confirm that IL-22 was a product of Th17 T lymphocytes, and was not expressed by the pancreatic cells. As expected, IL-22 mRNA expression did not change in CFA-treated NOD.Scid mice (data not shown).

### IL-22 treatment induces islet β-cell DNA synthesis *in vitro*

**Figure 5:**
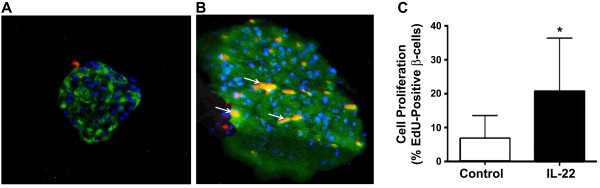
**Detection of pancreatic islet regeneration after IL-22 treatment.** Immunofluorescence localization of nuclear EdU and cytoplasmic insulin within pancreatic islets was done in 5 to 6-week-old NOD mice. Islets were treated with either DMEM media alone (***A***) or 10 ng/mL of recombinant IL-22 for 48 hrs (***B***) (red = EdU; green = insulin; blue = DAPI). Examples of dual-labeled cells are shown with arrows. The size bar = 50 μm. (***C)*** The mean percentage ± SEM of β-cells immunopositive for both EdU and insulin relative to insulin alone. Results were taken from 26 control islets and 46 IL-22-treated islets derived from 4 animals for each group. The asterisk (*) denotes a significant difference from the control (P<0.001). As described in the Methods section, in parallel experiments we explored the nuclear expression of Ki/67 as a measure of islet cell proliferation (data not shown). This further confirmed the proliferation of islet cells by IL-22 treatment.

To determine whether IL-22 can promote DNA synthesis in islet β-cells, we performed immunohistochemical analysis on isolated islets from 5 to 6-week-old NOD mice treated with recombinant IL-22 (10 ng/ml) for 48 hours and stained for nuclear EdU and cytoplasmic insulin (Figure 5A and B). The percentage of EdU positive β-cells was found to be significantly higher in islets treated with recombinant IL-22 when compared to control islets treated with media alone (P<0.001) (Figure 5C). To confirm results for DNA synthesis, slides containing IL-22 treated islets were stained for nuclear Ki/67 protein, which is associated with cell proliferation. The islets were stained with mouse anti-Ki/67 followed by incubation with secondary antibody. The positive expression of Ki/67 in the islets validated the induction of islet β-cells by IL-22 (data not shown). The ability of IL-22 to increase the percentage of β-cells undergoing DNA synthesis, as detected by nuclear labeling with EdU, was inversely related to islet size (y = −29logx + 56, r^2^ = 0.31, p<0.001, n=46). Islet size was estimated from the total number of insulin-immuno-reactive β-cells present per islet within tissue sections. No such relationship existed between islet size and the percent nuclear labeling of β-cells with EdU for control incubations (y = 2.2logx + 4.3, r^2^ = 0.01, non-significant, n=26). These findings suggest that the mitogenic actions of IL-22 on β-cells were greatest for the smaller islets.

## Discussion

We have previously shown that immunizing young NOD mice with CFA can cause regeneration of pancreatic β-cells by down-regulating autoimmunity and reducing insulitis [5, 11, 12]. However, the cellular mechanisms by which CFA is able to initiate a regenerative response are not well understood [7, 11]. Recently, CFA treatment in NOD mice has been shown to substantially up-regulate the regeneration gene, *Reg*2, in pancreatic islets during insulitis, thus functioning as an important regenerative candidate for β-cells [5]. The objective of this study was to determine whether the CFA-induced cytokine, IL-22, was in part responsible for inducing Reg2 expression and other gene family members, Reg1 and Reg3δ, leading to a subsequent increase in β-cell mass and reversal of T1D development.

**Figure 6:**
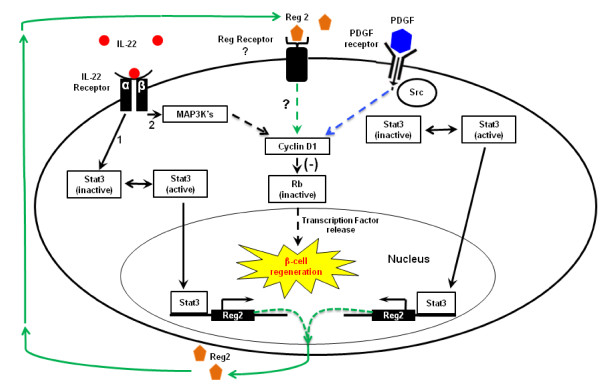
**A model for cytokine-mediated up-regulation of the** **β** **-cell** ***Reg*** **genes leading to** **β** **-cell regeneration.** Interleukin-22 (red) binds to its receptor complex, IL-22Rα/IL-10Rβ, which activates the Stat3 transcription factor protein (1), STAT3 then migrates into the nucleus of the β-cell and stimulates *Reg* gene transcription and translation (green). Secreted Reg proteins are then thought to activate Cyclin D1. This allows the β-cell to enter the G1/S transition of the cell cycle leading to regeneration. IL-22 can also activate the MAP3 kinases leading to Cyclin D1 (2), which in turn inactivates the Retinoblastoma (Rb). Platelet-derived growth factor (PDGF) (blue) similarly leads the activation of Cyclin D1, which in turn inactivates Rb allowing the release of sequestered transcription factors (TF) essential for the G1–S progression of the cell cycle. *Reg2* gene expression by activating Stat3, believed to be caused by receptor-induced Src kinase activity. The known signaling pathways (black and blue) and the proposed signaling pathways (green) are identified.

The exact mechanism underlying CFA-induced *Reg* gene upregulation has not been clearly defined. Experiments in the past have shown IL-6 to be an intermediate for *Reg*2 and *Reg*1 gene induction in the pancreas, since the IL-6 upstream response element is conserved among these *Reg* genes [5, 22]. Since IL-6 signaling leads to the activation of Stat3 transcription factors inside target cells [23], it was believed that *Reg*2 gene induction was also Stat-3 mediated [7]. Our results confirm and extend the role of the cytokine, IL-22, in up-regulating the *Reg*2 gene as well as *Reg1*, thus supporting our original hypothesis. We confirmed a 3-fold and 4.2-fold increase in *Reg*2 and *Reg*1 gene expression respectively when pre-diabetic (6-week-old) pancreatic islets were incubated with 10 ng/mL of recombinant IL-22 *in vitro* for 48 hrs. We have previously shown that the PCR results for the expression of Reg genes in the pancreatic islets in our studies correlate with the expression of Reg proteins by using Western blot assay [5]. This suggests that *Reg*2 gene expression, like *Reg*1, can be induced via Stat3 signaling and that IL-22 may be an immune response-mediated agent for the induction of these *Reg* genes within the pancreatic islets during insulitis [7, 16] (Figure 6). This finding is supported by a previous study by Aggarwal *et al.*[16], who had shown that the in vivo injection of IL-22 resulted in a rapid induction of *Reg2* expression in the pancreas of C57/BL6 mice. Interestingly, in our *in vitro* study recombinant IL-22 resulted in a noticeable down-regulation of *Reg2* and *Reg*1 expression in the islets from 6-week-old NOD mice when incubated with high concentrations (50 ng/mL) of IL-22. This may suggest that pancreatic β-cells exhibit a dose-dependent response to IL-22, and that an excess of the cytokine may be inhibitory to islet *Reg* gene expression. In contrast to the other *Reg* genes, the expression of *Reg*3δ was not affected by IL-22 treatment in the pancreatic islets and thus confirms past studies showing Reg3δ expression to be absent in the α- and β-cells. We also found *Reg*2 and *Reg*1 expression to be drastically reduced in the pancreas of pre-diabetic mice injected with a neutralizing IL-22 antibody, confirming that IL-22 is an upstream activator for these *Reg* genes. These findings support the study by Zhang *et al.*[24] who found the *Reg*1 and *Reg*2 genes to be induced by IL-22 in the colonic epithelial cells of *Citrobacter rodentium*-infected WT mice, but for expression to be completely abolished in IL-22 knock-out infected mice. As shown by our studies with Th17 supernatant, cytokines other than IL-22 may contribute to the upregulation of Reg genes. We are currently exploring this possibility in our laboratory.

Using the whole pancreas from NOD mice, Huszarik *et al.*[5] found several members of the *Reg* gene family to be up-regulated with age, and *Reg*2 to be further upregulated in diabetic mice following CFA treatment. Our results support the finding that *Reg*2 plays a dominant role in endogenous β-cell regeneration following adjuvant immunotherapy. *Reg*2 mRNA levels were found to gradually increase in pre-diabetic mice with age and were increased in diabetic mice following CFA immunization [5]. Similar to Reg2, IL-22 gene expression gradually increased with pre-diabetic age and was also increased following CFA treatment in both pre-diabetes and following the onset of diabetes. The similar pattern of expression between *Reg*2 and *IL-22* after CFA treatment further suggests *Reg*2 to be a gene target in the IL-22 signaling pathway. However, unlike IL-22, the expression of its receptor chain, IL-22Rα, unexpectedly dropped at all ages following CFA treatment. In a study analyzing mantle cell lymphoma growth caused by IL-22, Gelebart *et al.*[25] discovered that the gene promoter for IL-22Rα contained multiple binding sites for NF-κB, a transcription factor that when activated contributes to the pathogenesis and progressive loss of pancreatic β-cells by generating reactive oxygen species and promoting β-cell apoptosis. It is therefore possible that the effects of CFA may lead to the disruption or degradation of NF-κB that would in turn reduce/prevent IL-22Rα. Whether CFA affects the integrity of NF-κB, however, would require further investigation. Alternatively, IL-22 binding protein (IL-22BP) may increase in circulation with age and inhibit the expression of IL-22 receptor in pancreas.

In the spleen, we found *Reg* mRNA levels to be absent, thus supporting the finding by Huszarik *et al.*[5] that the *Reg* genes are pancreas-specific with/without CFA treatment. In contrast to *Reg*2 expression, however, the *IL-22* mRNA levels in splenocytes were found to be increased in non-diabetic (4-week-old) NOD mice following CFA injection, which confirms the finding by Nikoopour *et al.*[15] that IL-22 is a secretory product induced by CFA.

We have identified IL-22 as a *Reg* gene inducer in the NOD mouse pancreatic islets. Reg proteins act as autocrine/paracrine growth factor for pancreatic β-cell regeneration. They increase ATF-2 that binds to the cyclin D1 gene and leads to signaling pathway to induce β-cell regeneration [26]. It is also important to note, however, that like PDGF [10] and *Reg*1[9, 13], IL-22 has been shown by Radaeva *et al.*[27] to be directly involved in promoting cell cycle progression and cell survival. Using *in vitro* treatment of recombinant IL-22 at a concentration similar to our own study of 10 ng/mL, Radaeva *et al.*[27] were able to promote cell growth and survival of human hepatocellular carcinoma HepG2 cells via the activation of a series of anti-apoptotic and mitogenic proteins which included: Bcl-2, Bcl-xL, Mcl-1, c-myc, and cyclin D1. Due to the ontological similarity between hepatic cells and pancreatic cells, we also investigated the effect of IL-22 on pancreatic islet cell growth. We found increased DNA synthesis in the IL-22 treated islets. The extent to which this increase was dependent on the production of Reg2 or Reg1 by these islets, however, will require further investigation. The mitogenic effect of IL-22 was greater on the smaller-sized islets, suggesting that this population of β-cells may have a greater regenerative capacity. Smukler *et al.*[28] reported a sub-population of insulin-expressing β-cells located in small islets in both the mouse and human pancreas that had a high mitogenic potential, but did not express the glucose transporter-2. These cells were shown to be lineage multipotent and capable of forming multiple islet endocrine cell types, as well as neural cells, and may represent a β-cell progenitor population that can contribute to islet regeneration.

CFA-treated diabetic NOD mice have been previously shown to partially reverse T1D by down-regulating autoimmunity and stimulating endogenous β-cell regeneration through a mechanism that is presently unknown. This regeneration process, however, is not enough to prevent the disease entirely, as newly regenerated β-cells are still selectively destroyed by ongoing autoimmunity, but is believed to involve the mitogenic *Reg* gene family. We have demonstrated that the cytokine, IL-22, can up-regulate *Reg*2 and *Reg*1 expression when directly co-cultured with pre-diabetic pancreatic islets *in vitro*. Thus, IL-22 represents a newly discovered mediator for up-regulating islet *Reg* expression using a JAK/STAT3 signal transduction pathway. We have also shown that excess IL-22 may have an inhibitory effect on islet *Reg* expression, that the mRNA levels for IL-22 are up-regulated with increasing age and development of disease in the NOD mouse, that CFA treatment causes a down-regulation of the IL-22Rα receptor chain, and that IL-22 can increase β-cell DNA synthesis when directly applied to pancreatic islets *in vitro.*

In conclusion, this study has identified the cytokine, IL-22, to be involved in up-regulating *Reg*2 and *Reg*1 gene family members and increasing β-cell DNA synthesis within pancreatic islets. This could result in β-cell regeneration in islets and that could potentially be incorporated into future therapeutic strategies for disease management in Type 1 diabetes.

## Methods

### Animals

Female NOD/Ltj mice at 4, 8 and 12 weeks of age, and diabetic NOD mice, shortly after the diagnosis of diabetes (blood glucose greater than 11 mM and polyuria), as well as NOD.SCID mice at 4 weeks or as adults, were obtained from the University of Western Ontario, London, Ontario, Canada and housed in a pathogen-free environment. Mice were housed three per cage, with food and water provided ad libitum, and maintained on a 12-hour light/dark cycle. Blood glucose was measured from a tail sample using a glucometer (Bayer, Elkhart, IN). All experiments followed the Canadian Council for Animal Care guidelines and were approved by the Animal Use Sub-Committee, University of Western Ontario.

### Treatments

Complete Freund’s adjuvant (CFA) was purchased from Sigma Aldrich (St. Louis, MO). To determine the effect of CFA on *Reg*2, *Reg*1, *Reg*3δ, *IL-22*, and *IL-22-receptor(R)-*α expression, mice were injected i.p. either with 100 μl of CFA (50 μg/mL) emulsified in saline (1:1), or with 100 μl of saline alone for control in 4, 8, 12-week, and diabetic NOD mice. To determine the effect of IL-22 neutralization on *Reg* expression; three 5 to 6 week-old NOD mice were injected i.p. with 100 μl of CFA with saline (1:1) followed 1 hr later with 100 μl (i.p.) of 3 mg/mL rat anti-mouse IL22-01 monoclonal antibody (21) or isotype matched control antibody (Pfizer). Mice were sacrificed in a CO_2_ chamber 48 hrs later for tissue extraction.

### Extraction of splenocytes and pancreas

Approximately 50 mg of whole spleen or pancreatic tissue from NOD/Ltj and NOD.SCID mice was homogenized in a solution containing buffer RLT (Qiagen, Mississauga, ON) and 2-Mercaptoethanol (Sigma Aldrich, St. Louis, MO) using a PowerGen 700 homogenizer (Fisher Scientific, Pittsburgh, PL).

### Isolation of pancreatic islets

NOD mice were sacrificed by cervical dislocation. Cold collagenase XI (0.23 mg/mL, Sigma Aldrich, St. Louis, MO) was directly injected into the pancreas through the common bile duct. Collagenase at 37°C was used to digest the pancreatic tissue. Connective tissues and remaining cells were removed using Hank’s buffered salt solution (HBSS) (Invitrogen, Carlsbad, CA) containing 5% (v/v) fetal calf serum (FCS) (HyClone Laboratories, Logan, UT). Islet separation was accomplished by density gradient centrifugation using dextran (Sigma Aldrich, St. Louis, MO). Cells located at the interface of dextran gradient layers of 11% and 23% were harvested by pipette and washed in Roswell Park Memorial Institute (RPMI) 1640. Individual pancreatic islets were handpicked and cultured in Dulbecco’s modified eagle medium (DMEM) (Invitrogen, Carlsbad, CA) supplemented with 100 U/mL streptomycin (GIBCO, Grand Island, NY), 5 μg/mL penicillin, and 10% (v/v) FCS at 37°C.

### Culture of pancreatic islets cells with IL-22

Pancreatic islets were isolated from young (4–6 wk old) NOD mice. They were equilibrated overnight at 37°C, 5% CO_2_ in RPMI-1640 medium supplemented with 10% fetal calf serum (FCS), 100 U/mL streptomycin and 5 μg/mL penicillin and islets were handpicked and distributed into 24 well plate (~50 islets per well) and treated with recombinant mouse IL-22 (10 ng/ml or 50 ng/ml, R&D systems, Minneapolis, MN) or with 2 ml of Th17-cell supernatant from IL-6/IL-23 treated splenocytes [15]. Two days later, islets were harvested for RNA extraction.

### RNA extraction

Total RNA from isolated splenocytes, pancreatic or islet cells was extracted from homogenates using an RNeasy Midi Kit (Qiagen, Mississauga, ON). A DNase 1 treatment kit (Ambion, Austin, TX) was used to eliminate DNA contamination. The RNA content was quantified by measuring absorbance at 260 nm using a Nanodrop 1000 spectrophotometer (NanoDrop Products, Wilington, DL), and the integrity of selected RNA samples was checked using agarose gel electrophoresis and ethidium bromide to identify the presence of the 18S and 28S rRNA bands.

### Real time polymerase chain reaction

Approximately 1 to 5 μg of total RNA was taken from each sample and reverse transcribed into cDNA using oligo dT_12-18_ primers from Superscript III first-strand Synthesis SuperMix for quantitative RT-PCR (Invitrogen, St. Louis, MO). The cDNA concentration was measured using a spectrophotometer and was diluted to 225 ng/μL in diethyl pyrocarbonate water and amplified by PCR using a Quantifast SYBR Green PCR Kit (Qiagen, Mississauga, ON) with specific primers for *Reg*1, *Reg*2, *Reg*3δ, *IL-22* and *IL-22*Rα1.25 μL) (Table 1). DNA was amplified by PCR in a two-step melting/annealing program for up to 40 cycles using a Corbett Rotor-Gene 6000 thermocycler (Corbett Life Sciences, San Francisco, CA). This thermocycler program consisted of preheating the samples at 95°C for 5 min, cycling at 95°C for 10 sec and 60°C for 20 sec, and a melting phase of 60 to 95°C. β-actin was used as the housekeeping gene for relative quantification. The efficiency of each set of primers was determined by the standard curve method.

**Table 1:** **List of genes and primer sequences used for qRT-PCR**

Gene symbol	Forward primer (5’→3’)	Reverse primer (5’→3’)	Product size (bp)
*Reg*2	cactgccaaccgtggttat	gacaaaggagtactgtgcctca	75
*Reg*1	catctgccaggatcagttgc	aggtaccataggacag	549
*Reg*3δ (INGAP)	ccatggtgtctcacaagacc	tgatgcgtggagaagacagt	117
*IL-22*	tcagctcagctcctgtcacat	tccccaatcgccttgatctct	117
*IL-22R*α	gctcgctgcagcacactacca	tctgtgtcgggagtcaggcca	247
β*-actin*	gcccagagcaagagaggtat	cacacgcagctcattgtaga	116

### Fluorescence immunohistochemistry for detection of β-cell DNA synthesis

Approximately 50 isolated islets from 5 to 6 week-old NOD mice were added to four wells of a six well tissue culture plate and treated either with 10 ng/mL of recombinant IL-22 as above in DMEM media, or in DMEM media alone as a control, for 48 hrs. Islets were fixed in 4% paraformaldehyde (PFA) for 1 hr at 4°C and re-suspended in 2% low melting point (LMP) Agarose (Fisher Scientific) which was first liquefied by heating to 80°C. The islets embedded in Agarose were dehydrated in 70% ethanol and embedded in paraffin. Sections of 5 μm were cut and mounted on glass slides. EdU staining was performed using a Click-it EdU cell proliferation kit (Invitrogen) according to the manufacturer’s instructions. Non-specific staining was blocked using Background Sniper (Biocare Medical, Concord, CA, U.S.A.) for 10 min, and the sections incubated overnight with the primary antibody, mouse anti-insulin (1:2000; Sigma Aldrich, St. Louis, MO) followed by a 1 h incubation with secondary antibody, Alexafluor 488 goat anti-mouse (1:500; Invitrogen). Cells were co-stained with nuclear DAPI (1:500; Sigma Aldrich, St. Louis, MO). Twenty-six control and forty-six treated islets were identified and analyzed, and the percentage of EdU-positive β-cells was quantified. To confirm results for DNA synthesis, slides containing sections from 15 additional islets were stained for nuclear Ki/67 overnight using the primary antibody, mouse anti-Ki/67 (1:2000; Sigma Aldrich), followed by a 1 h incubation with secondary antibody, Alexafluor 555 goat anti-mouse (1:500; Invitrogen). Analysis of the islet sections was performed by fluorescence microscopy and the images were analyzed using Northern Eclipse Version 7.0 software (Empix Imaging, Inc., Mississauga, ON, Canada).

### Statistical analysis

Normally distributed data were analyzed with SPSS statistical software using a one-way or two-way ANOVA, and significant changes between variables were detected using a Tukey’s test. The α value was set to 0.05.
